# Formal commentary

**DOI:** 10.1371/journal.pgen.1009059

**Published:** 2020-11-05

**Authors:** Gustavo D. Aguirre, Hannes Lohi, Maria Kaukonen, Leonardo Murgiano

**Affiliations:** 1 School of Veterinary Medicine, University of Pennsylvania, Philadelphia, Pennsylvania, United States of America; 2 Department of Medical and Clinical Genetics, University of Helsinki, Helsinki, Finland; HudsonAlpha Institute for Biotechnology, UNITED STATES

Two recent papers by Gustavo Aguirre’s and Hannes Lohi’s groups have been published describing the genetics of Progressive Retinal Atrophy (PRA) in Miniature Schnauzers. This recessively inherited condition is a serious problem in companion animals, and also is a model for human retinitis pigmentosa in humans. The first paper by Murgiano et al. [1] proposed a complex structural variant affecting the coding region of the PPT1 gene as causal, but some dogs were apparently homozygous for the variant and did not exhibit disease, suggesting incomplete penetrance. A more recent study by Kaukonen et al. [2] identified the same locus, but identified a different candidate causal variant—a SNV in an intron of the HIVEP3 gene, which lies ~1.5 Mbp away from PPT1. In the Kaukonen et al. study, the candidate causal variant appeared completely penetrant.

The different conclusions drawn in the two published papers have facilitated discussions and comparisons of the datasets between the groups, and some re-analyses have been conducted to test the presented alternate hypotheses of the causal candidate genes and variants. Importantly, these re-analyses reveal new insights which affect the interpretation of the data and related conclusions. We take this opportunity to summarize our analyses in a joint statement.

Key observations affecting the interpretations and conclusions in both studies relate to: (1) discovery of the non-mutated risk haplotype as described by Kaukonen et al.; and (2) the realization that is challenging to determine the zygosity of the complex PPT1 structural variant without high quality WGS data.

In the work from Kaukonen et al., a heterozygous obligate carrier dog was found to be homozygous for SNV markers previously described by Murgiano et al. as associated with the condition, revealing that the PRA mutation is recent, with both mutant and non-mutant chromosomes with the same haplotype segregating in Miniature Schnauzers. This same obligate carrier was originally inferred to be homozygous for the PPT1 structural variant based on SNV genotyping; however, a new coverage-based analysis of WGS data revealed that the dog is actually heterozygous for the PPT1 structural variant (and is also heterozygous for the HIVEP3 variant).

Thus, the genetic evidence is unable to distinguish between the HIVEP3 and PPT1 variants as potential causes of PRA. Furthermore, the SNV markers initially proposed in Murgiano et al. are not reliable to determine the zygosity of the complex PPT1 structural variant, or to reach conclusions regarding its potential penetrance. Although functional considerations favor causality of the coding PPT1 structural variant over the intronic HIVEP3 SNV, an efficient and inexpensive means of genotyping the PPT1 structural variant has not been developed, limiting our ability to reach final conclusions about the true causal gene. It is also possible that PRA is caused by a different variant in the mapped interval that could not be identified by whole genome sequencing.

For the purposes of diagnosis by breeders and veterinarians, the HIVEP3 variant may be used in a genetic testing environment until final conclusions of the causal variant are made and a robust method to genotype the PPT1 structural variant becomes available. Importantly, when testing the HIVEP3 variant one should bear in mind that if HIVEP3 is not the causal gene, recombination between HIVEP3 and the causal variant would produce incorrect test results.
